# Procoagulant Extracellular Vesicles Alter Trophoblast Differentiation in Mice by a Thrombo-Inflammatory Mechanism

**DOI:** 10.3390/ijms22189873

**Published:** 2021-09-13

**Authors:** Paulina Markmeyer, Franziska Lochmann, Kunal Kumar Singh, Anubhuti Gupta, Ruaa Younis, Khurrum Shahzad, Ronald Biemann, Hanna Huebner, Matthias Ruebner, Berend Isermann, Shrey Kohli

**Affiliations:** 1 Institute of Laboratory Medicine, Clinical Chemistry and Molecular Diagnostics, University Hospital Leipzig, Leipzig University, 04103 Leipzig, Germany; paulina.markmeyer@web.de (P.M.); franziskalochmann@freenet.de (F.L.); Kunal.Singh@medizin.uni-leipzig.de (K.K.S.); Anubhuti.Gupta@medizin.uni-leipzig.de (A.G.); Ruaa.Younis@medizin.uni-leipzig.de (R.Y.); Khurrum.Shahzad@medizin.uni-leipzig.de (K.S.); ronald.biemann@medizin.uni-leipzig.de (R.B.); 2 Institute of Clinical Chemistry and Pathobiochemistry, Otto-von-Guericke-University Magdeburg, 39120 Magdeburg, Germany; 3 Department of Gynecology and Obstetrics, University Hospital Erlangen, Friedrich-Alexander-University Erlangen-Nuremberg, 91054 Erlangen, Germany; hanna.huebner@uk-erlangen.de (H.H.); matthias.ruebner@uk-erlangen.de (M.R.)

**Keywords:** thrombo-inflammation, extracellular vesicles, inflammasome, trophoblast differentiation

## Abstract

Procoagulant extracellular vesicles (EV) and platelet activation have been associated with gestational vascular complications. EV-induced platelet-mediated placental inflammasome activation has been shown to cause preeclampsia-like symptoms in mice. However, the effect of EV-mediated placental thrombo-inflammation on trophoblast differentiation remains unknown. Here, we identify that the EV-induced thrombo-inflammatory pathway modulates trophoblast morphology and differentiation. EVs and platelets reduce syncytiotrophoblast differentiation while increasing giant trophoblast and spongiotrophoblast including the glycogen-rich cells. These effects are platelet-dependent and mediated by the NLRP3 inflammasome. In humans, inflammasome activation was negatively correlated with trophoblast differentiation marker GCM1 and positively correlated with blood pressure. These data identify a crucial role of EV-induced placental thrombo-inflammation on altering trophoblast differentiation and suggest platelet activation or inflammasome activation as a therapeutic target in order to achieve successful placentation.

## 1. Introduction

Proper placentation is required for a successful pregnancy outcome. Vascular complications resulting in placental dysfunction, also referred to as gestational vascular diseases (e.g., pre-eclampsia, HELLP), are a leading cause of pregnancy failure and feto-maternal morbidity and mortality. Physiologically, the hemostatic, as well as the immune system, are altered during pregnancy. While the physiology of these changes remains incompletely defined, it is established that pathological changes of these systems promote placental dysfunction and adverse pregnancy outcomes, potentially by impeding trophoblast differentiation.

Successful placentation depends on proper trophoblast cell differentiation in humans and mice. Placentation in humans is different from that of mice. However, they both share several similarities, which are not available in other animal or rodent models [1]. As systematic studies are not possible in humans, the mouse model has been instrumental for systematic analyses and advancing our knowledge of trophoblast differentiation and placentation in vivo. In mice, the placenta consists of the maternal decidua basalis (DB) and the embryonic part of the placenta consisting of the trophoblast. The trophectoderm cells endoreduplicate thereby forming the primary trophoblast giant cells which help in implantation. Trophoblast cells differentiate into three major layers—the (i) giant trophoblast, the spongiotrophoblast zone (SZ), together comprising the junctional zone (JZ), and the labyrinth zone (LZ) including the syncytiotrophoblast [2,3]. Primary giant trophoblast cells arise from the trophectoderm and mediate implantation. After implantation, the cells of the ectoplacental cone differentiate into secondary giant trophoblast cells which invade the decidua. Additionally, the ectoplacental cone gives rise to the spongiotrophoblast layer (also called the junctional zone). In the mature placenta, this forms the middle layer between the giant trophoblast cells and the labyrinth zone. Glycogen trophoblast cells appear within the spongiotrophoblast starting at day 12.5 p.c. Chorioallantoic fusion, which begins at around E8.5, results in the formation of the vascular labyrinth which includes the syncytiotrophoblast [2,3]. Transcription factor GCM1 plays an important role in the formation of syncytiotrophoblast and the labyrinth. Proper development of the placental vascular labyrinth is required for feto-maternal transport. Defective development of the labyrinth is a frequent cause of developmental failure and growth deficits that often occur mid-gestation [2]. A key feature of the labyrinth layer of the mouse placenta is the direct contact of mouse trophoblast cells with maternal blood—a characteristic that is also present in human placental villi, thus providing direct access to blood-derived components, such as coagulation regulators, to trophoblast cells.

Trophoblast differentiation and proliferation are in part regulated by coagulation regulators, some of which are expressed in the placenta. Pathological coagulation activation, e.g., excess platelet activation, is associated with impaired placentation. Procoagulant extracellular vesicles (EVs) promote platelet activation and have been linked with vascular complications in pregnancy. We have previously shown that EVs cause preeclampsia by a thrombo-inflammatory mechanism involving activation of the placental NLRP3 inflammasome. Of note, the NLRP3 inflammasome has been linked with cell-differentiation in non-trophoblast cells [4,5], which raises the question as to whether EVs and platelet activation impairs trophoblast differentiation via excess NLRP3 inflammasome activation, thus contributing to placental dysfunction. Aspirin effectively inhibits COX1 and thus thromboxane synthesis and platelet activation [6]. Platelet inhibition using aspirin has been shown to have a beneficial effect in patients with or at risk of developing preeclampsia (PE) [7]. We have previously shown that platelet inhibition using aspirin prevents EV-induced thrombo-inflammation at the maternal–fetal interface, which is linked to inflammasome activation in trophoblast cells [8]. Hence, inhibition of IL-1R signaling, and its effect on trophoblast differentiation using Anakinra, may offer an alternative therapeutic intervention in PE. To address this question, we determined the role of the platelet-dependent NLRP3 inflammasome on trophoblast differentiation and placental morphogenesis.

## 2. Results

### 2.1. Extracellular Vesicles and Platelets Alter Trophoblast Morphology, Differentiation, and Glycogen-Rich Cells

**Figure 1:**
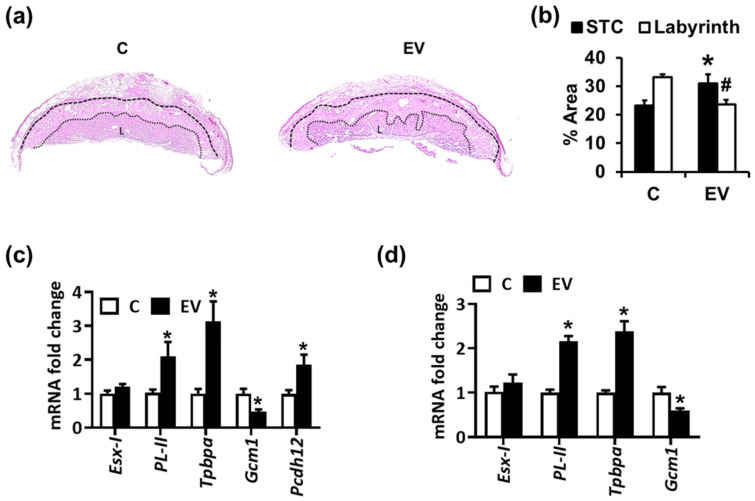
Extracellular vesicles (EVs) alter placental morphology and trophoblast differentiation. (**a**,**b**) Hematoxylin and eosin (H&E) stained placentae ((**a**), representative images; (**b**), bar graph summarizing results) showing a reduced labyrinth zone (LZ) and increased junctional zone (JZ) in placenta from EV-injected mothers. * *p* < 0.05 (STC), # *p* < 0.05 (labyrinth); *t*-test. (**c**,**d**) qPCR analysis for markers of trophoblast differentiation and glycogen-rich cells in mouse placenta (**c**) or mouse trophoblast stem cells (**d**) showing an increase in spongiotrophoblast marker (*Tpbpa*), giant trophoblast marker (*PL-II*) and glycogen-rich cell marker (*Pcdh12*), while the marker for syncytiotrophoblast differentiation *Gcm-1* decreases upon EV treatment (**c**,**d**). * *p* < 0.05; *t*-test (**c**,**d**). *n* = 6 placentae from 3 different litters each group (**a**–**c**) or 3 independent repeat experiments (**d**).

In order to determine the effect of EV-mediated thrombo-inflammation on placental development, we first studied the gross morphology of the placenta by determining the proportion of spongiotrophoblast (STC) and the labyrinth zone. In placentae from mothers injected with procoagulant EVs, the border between the STC and LZ was irregular, and the relative area of the STC was increased, while that of the labyrinth was reduced (Figure 1a,b). This indicates an abnormal differentiation of the trophoblast and developmental delay of the labyrinth. Based on RT-PCR analysis of trophoblast differentiation marker genes, EV administration enhanced giant trophoblast (*PL-II*) and spongiotrophoblast (*Tpbpa*) differentiation as compared to PBS-injected control pregnant mice. Glycogen-rich cells (GRCs) present within the STCs constitutes the placental glycogen stores providing a source of glucose to support fetal growth (Figure 1c). *Pcdh12*, a specific GRC marker, was increased, indicating increased GRCs within this zone (Figure 1c). On the other hand, the syncytiotrophoblast differentiation marker, *Gcm-1*, was reduced upon EV administration (Figure 1c). These findings indicate dysregulated trophoblast differentiation as a cause of altered placental development due to procoagulant EVs. In vitro experiments with differentiating mouse trophoblast stem cells (mTS) exposed to EVS and platelets revealed similar findings, except for GRCs, which do not form in this in vitro model (Figure 1d).

**Figure 2:**
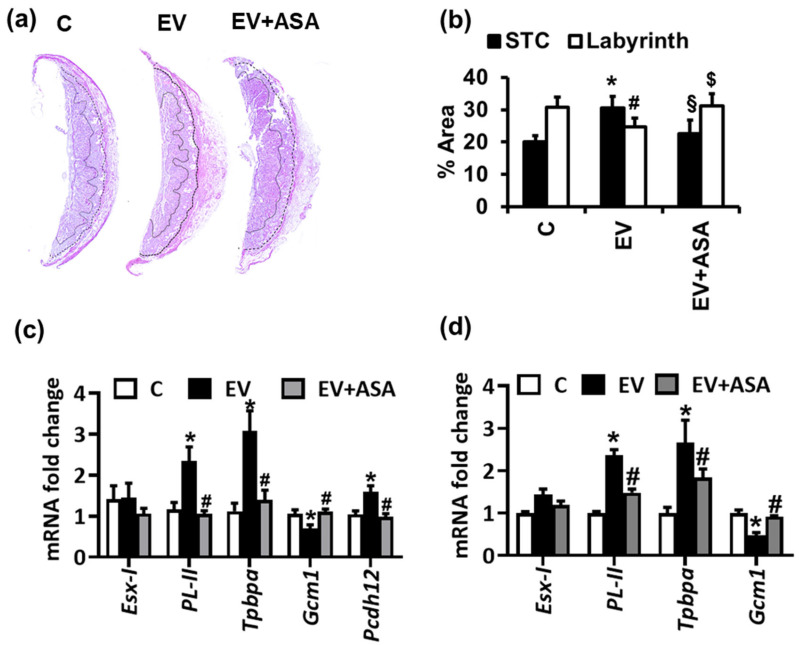
Platelet inhibition using aspirin prevents EV-induced impaired trophoblast differentiation. (**a**,**b**) Hematoxylin and eosin (H&E) stained placentae. (**a**), representative images; (**b**), bar graph summarizing results) showing restoration of labyrinth zone (LZ) and junctional zone (LZ) relative area in placenta from EV-injected mothers upon aspirin treatment. * *p* < 0.05 (STC, relative to C), # *p* < 0.05 (labyrinth, relative to C), § *p* < 0.05 (STC, relative to EV), $ *p* < 0.05 (labyrinth, relative to EV); ANOVA. (**c**,**d**) qPCR analysis for trophoblast differentiation markers in mouse placenta (**c**) or mouse trophoblast stem cells (**d**) showing that inhibition of platelet activation by aspirin prevents the EV-induced increase in spongiotrophoblast marker (*Tpbpa*), giant trophoblast marker (*PL-II*) and glycogen-rich cell marker (*Pcdh12*). In parallel, aspirin prevents the EV-induced decrease in syncytiotrophoblast marker *Gcm-1* in mice (**c**) or trophoblast cells (**d**). * *p* < 0.05 (relative to C), # *p* < 0.05 (relative to EV); ANOVA (**c**,**d**). *n* = 6 placentae from 3 different litters each group (**a**–**c**) or 3 independent repeat experiments (**d**).

Since procoagulant EVs can activate platelets in vivo [8,9,10], we next evaluated whether the altered histology and trophoblast differentiation, seen upon EV injections, depends on platelet activation. Platelet inhibition using aspirin treatment prevented the histological changes and altered expression of trophoblast differentiation marker genes and of glycogen-rich cells observed upon EV administration (Figure 2a–c). Similar to the in vivo situation, platelets from aspirin-injected mice did not impair the trophoblast differentiation marker gene expression in vitro in EV-exposed differentiating murine trophoblast stem cells (Figure 2d). Taken together, these results suggest that the effects of EVs on trophoblast differentiation and glycogen-rich cells depend on platelet activation.

### 2.2. NLRP3-Mediated Thrombo-Inflammation Causes EV-Induced Altered Trophoblast Differentiation and Glycogen-Rich Cells

**Figure 3:**
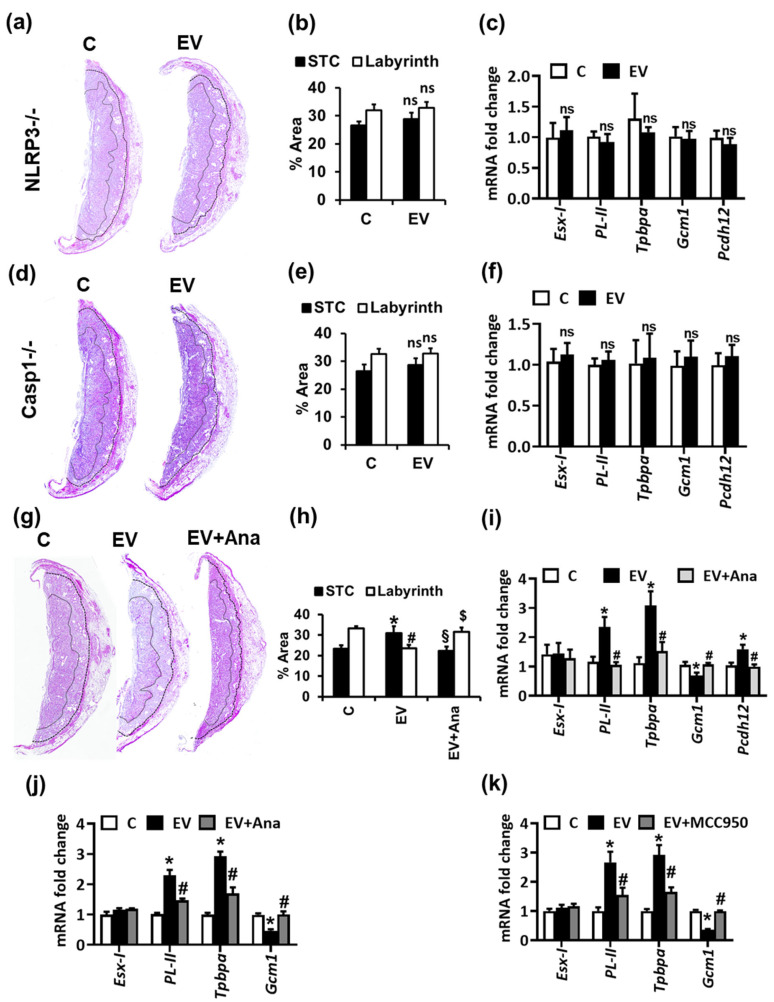
EVs impair trophoblast differentiation in an inflammasome-dependent manner. (**a**–**f**) NLRP3 or Caspase-1 knockout prevents EV induced trophoblast alterations. Hematoxylin and eosin (H&E)-stained placentae ((**a**,**d**), representative images; (**b**,**e**), bar-graph summarizing results) show that NLRP3^−/−^ (**a**,**b**) or Casp-1^−/−^ (**d**,**e**) mice are protected from EV-induced morphological alterations in junctional and labyrinth zone. qPCR analysis for trophoblast differentiation markers (**c**,**f**) in NLRP3^−/−^ or Casp-1^−/−^ mouse placenta showing no change in differentiation markers (*Tpbpa*, *PL-II*, *Gcm-1*) or glycogen-rich cell marker (*Pcdh12*) upon EV treatment. ns: non-significant; ANOVA (**b**,**c**,**e**,**f**). (**g**–**k**) Inflammasome inhibition prevents EV-induced trophoblast alterations. In vivo intervention with the IL-1R antagonist anakinra in EV injected pregnant mice prevents morphological alterations (H&E staining; (**g**), representative images; (**h**), bar graph summarizing results) of labyrinth zone (LZ) and junctional zone (JZ) in placenta from EV-injected mothers. * *p* < 0.05 (JZ, relative to C), # *p* < 0.05 (LZ relative to C), § *p* < 0.05 (JZ, relative to EV), $ *p* < 0.05 (LZ relative to EV); ANOVA (**h**). (**i**–**k**) qPCR analysis for trophoblast differentiation and glycogen-rich cell markers in mouse placenta (**i**) or mouse trophoblast stem cells (**j**,**k**) showing that inflammasome inhibition using anakinra (**i**,**j**) or the NLRP3 inhibitor MCC950 (**k**) prevents the EV-induced increase in spongiotrophoblast marker (*Tpbpa*), giant trophoblast marker (*PL-II*) and glycogen-rich cell marker (*Pcdh12*), while reversing the EV-induced suppression of the syncytiotrophoblast marker *Gcm-1*. * *p* < 0.05 (relative to C), # *p* < 0.05 (relative to EV); ANOVA (**i**–**k**). *n* = 6 placentae from 3 different litters each group (**a**–**i**) or 3 independent repeat experiments (**j**,**k**).

EV-induced platelet activation triggers ATP-mediated purinergic receptor signaling and NLRP3 inflammasome activation within the placenta [8]. Therefore, we studied whether pharmacological or genetic inflammasome inhibition can prevent the effects of EVs on placental development. EV administration failed to alter the thickness and morphology of STCs or labyrinth in NLRP3^−/−^ as well as in Casp-1^−/−^ mice in comparison to WT (C57BL/6) mice (Figure 3a–f). Furthermore, we did not observe any significant difference in the trophoblast differentiation marker genes in these mice upon EV injections (Figure 3c,f). Similarly, treatment of EV-injected WT pregnant female mice with IL-1R antagonist anakinra restored the morphological alterations, impaired trophoblast differentiation, and GRCs (Figure 3g–i). Anakinra treatment or NLRP3 inhibition using MCC950 in EV-treated mTS cells likewise prevented EV-induced altered trophoblast differentiation (Figure 3j,k). These findings indicate that EV-induced platelet-mediated inflammasome activation in the murine placenta and trophoblast cells impair trophoblast differentiation and GRCs within the placenta.

### 2.3. Trophoblast Differentiation Is Correlated with Placental Thrombo-Inflammation in PE Patients

**Figure 4:**
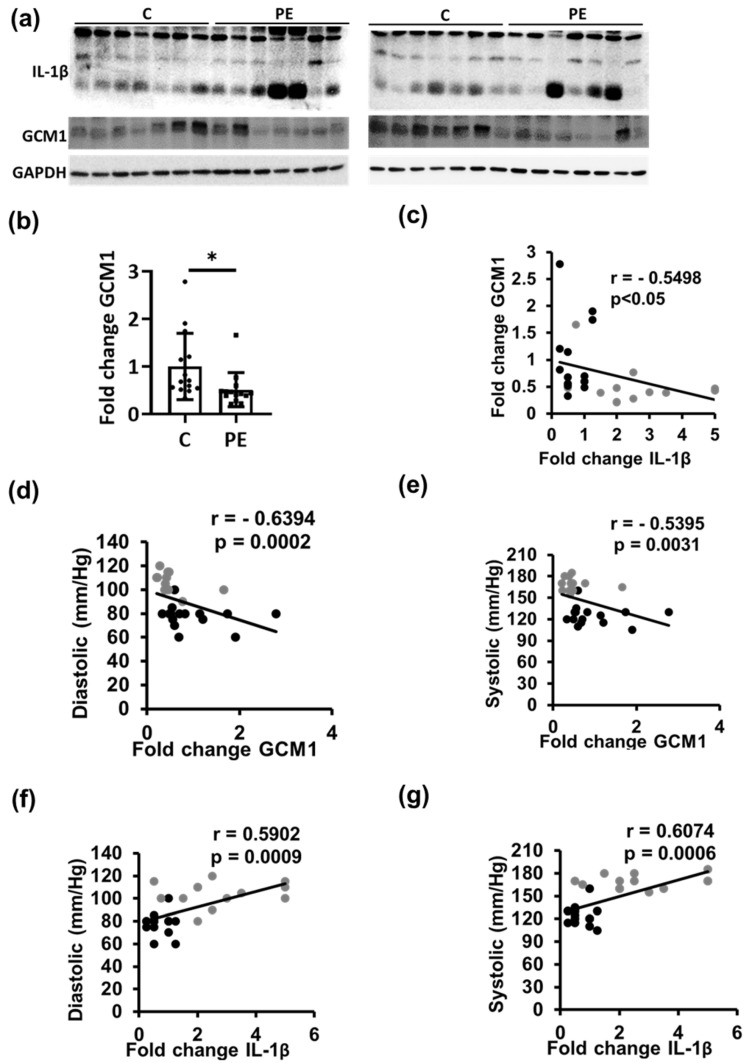
Trophoblast differentiation is associated with inflammasome activation in human PE placenta. (**a**–**c**) Trophoblast differentiation marker GCM1 (**a**), representative immunoblots; (**c**,**d**), quantification summarizing results) is reduced (**c**) in human placenta from preeclampsia (PE) and inversely correlated with inflammasome marker cleaved IL-1β (**b**) in control (C, black dots) and PE (grey dots) patients. (**d**–**g**) Diastolic and systolic blood pressure are negatively correlated with trophoblast differentiation marker GCM1 and positively correlated with inflammasome marker (IL-1β) in control and preeclampsia patients. *n* = 14 placenta each group; * *p* < 0.05 (correlation: *t*-test, (**c**–**g**): Spearman’s correlation).

In order to corroborate our findings in the human placenta, we studied the trophoblast differentiation marker GCM1 in placentae from preeclamptic and healthy pregnant women (Table S1). In humans, GCM1 is an important regulator of trophoblast cell differentiation along both villous and extra-villous pathways [11]. Reduced placental GCM1 expression causes defective syncytiotrophoblast differentiation and maternal and placental preeclampsia-like phenotypes in mice [12] and is inversely associated with placental vascularization in humans [12]. These data support a crucial function of Gcm1 for the development of a hemochorial placenta. In our study, Gcm-1 expression in human placentae was reduced in preeclampsia patients compared to healthy controls and negatively correlated with inflammasome marker cleaved IL-1β. Furthermore, blood pressure, a clinical parameter used to identify the onset of preeclampsia, was negatively correlated with GCM1 and positively correlated with IL-1β expression (Figure 4). This indicates that an enhanced inflammasome activation, likely due to platelet activation, is associated with impaired trophoblast differentiation in humans.

## 3. Methods

### 3.1. Mice

Wild-type C57BL/6, caspase-1^−/−^ and NLRP3^−/−^ mice were obtained from The Jackson Laboratory, USA [8,13]. The knockout mice were crossed onto the C57BL/6 background for at least nine generations. Pregnant mice were injected with EVs at days 10.5 and 11.5 p.c., as described earlier [8,14]. Anakinra or aspirin was injected intraperitoneally 30 min prior to each EV injection [8,15]. All animal experiments were conducted following standards and procedures approved by the local Animal Care and Use Committee (Landesverwaltungsamt, Halle, Germany).

### 3.2. Generation of Procoagulant EVs

EVs were prepared, quantified for procoagulant potential using ZYMUPHEN™ MP-activity assay, and used using previously described methods [8,14]. Mouse-derived SVEC cells (mouse endothelial cells, ATCC) were serum-starved for 72 h to generate EVs. Cell culture supernatant was collected, centrifuged at 200× *g* for 10 min, followed by high-speed centrifugation at 20,000× *g* for 45 min to pellet endothelial cell-derived EVs. After centrifugation, the EV pellet was washed twice with PBS followed by centrifugation at 20,000× *g* for 45 min each time. PBS was centrifuged at the same conditions before the wash. The EV pellet was finally re-suspended in PBS, aliquoted, and stored at −800 °C until further use. Supernatant from the previous wash was centrifuged at 20,000× *g* for another 40 min and used as a control for all experiments. As we intended to mimic the procoagulant activity of EVs, independent of other properties, we assessed the procoagulant activity (thrombin generation potential in nM) of EVs (thawed once) as well as the supernatant of the last wash which was assessed using Zymuphen MP-Activity ELISA. EVs used for experiments were obtained from the same lot and were likewise only thawed once in order to maintain consistency. All EVs were and used within 1 month of preparation. The size of EVs was identified using FACS analysis using size-based beads and was found to correspond to 1 μm size beads indicating that the vesicle fraction largely included microvesicles. EV concentration was adjusted to 600 nM pro-coagulant activity per kg bodyweight (nM/kg bodyweight) before injection. In all experiments, EVs of different cellular origin were used separately.

### 3.3. Cell Culture

To generate platelet-rich plasma (PRP), citrated blood was centrifuged at 160× *g* for 20 min [8]. Mouse trophoblast stem (TS) cells were obtained from J. Rossant (Hospital for Sick Children, Toronto, ON, Canada) and were maintained as stem cells or induced to differentiate [8,16,17]. Standard (stem cell maintenance) TS cell medium consisted of 20% FBS, 1 mM sodium pyruvate, 50 µg/mL penicillin/streptomycin, 5.5 × 10^−5^ M β-mercaptoethanol, 25 ng/mL basic fibroblast growth factor and 1 µg/mL heparin in RPMI 1640. Seventy percent of this medium was preconditioned by incubation on embryonic fibroblasts for 48 h. Differentiating TS cell medium consisted of 20% FBS, 1 mM sodium pyruvate, 50 µg/mL penicillin/streptomycin, and 5.5 × 10^−5^ M β-mercaptoethanol in RPMI 1640. The cells were routinely characterized for expression of markers of undifferentiated and differentiated TS-cells using RT-PCR [16]. mTS cells differentiated for 4 days were treated with EV (final procoagulant activity: 7.5 nM thrombin equivalent) and PRP for 24 h in differentiation medium. In some experiments, cells were exposed to anakinra (5 μg/μL) or MCC950 (10 nM) along with EV and PRP or to EV and PRP obtained from aspirin (10 mg/kg)-injected mice. In order to obtain PRP from aspirin-injected mice, the mice were injected with 10 mg/kg aspirin for three consecutive days and blood was obtained 1 h after the last dose from the vena cava.

### 3.4. Human Samples

Human placenta samples used in the study were collected from the University Hospital Erlangen. All of the participants provided written informed consent, and the medical faculty’s ethics committee of Friedrich-Alexander-University Erlangen-Nuremberg (No. 353_15 B) approved the study.

### 3.5. Immunoblotting

Gcm-1 (Biorbyt, Cambridge, UK) and IL-1β (Boster, Pleasanton, CA, USA; distributed by Biozol, Eching, Germany) primary antibodies and anti-rabbit HRP secondary antibodies were used in the study. Immunoblotting was conducted as previously described [14,18].

### 3.6. RNA Isolation and qRT-PCR

Total RNA was isolated using TRIzol reagent (Ambion, Thermo Scientific, Bremen, Germany) and RNA quality was assessed using gel-electrophoresis. Reverse-transcription was performed by the RevertAid First Strand cDNA Synthesis Kit (Thermo Scientific, Bremen, Germany). Quantitative polymerase chain reaction was performed in a Bio-Rad real-time system (CFX-Connect, Bio Rad, Hercules, CA, USA) using SYBR Green (Thermo Scientific, Bremen, Germany). The mRNA levels of the genes tested were normalized to GAPDH as an internal control. The primer sequences were:

Mouse Gcm1: Forward 5′-GCTCTTGTGGCCCGAGTTC-3′ and Reverse 5′-GTTTTCACGTTCTGAGGCAGTT-3′; Mouse Esx1: Forward 5′-CATCTGCTTCACCCCGATCC-3′ and Reverse 5′-TCTGAAACCAAACCTGCACTCT-3′; Mouse Tpbpa: Forward 5′-GAAATGAGTGCCTCCGGTCA-3′ and Reverse 5′-TGTCCATGTTACTGTGGCTGATT-3′; Mouse PL-II: Forward 5′-CCAACGTGTGATTGTGGTGT-3′ and Reverse 5′-TCTTCCGATGTTGTCTGGTGG-3′; Mouse Pcdh12: Forward 5′-CTGTTGGACCCTAATACAGGTCT-3′ and Reverse 5′-TGCCGAATGTCTGGAAGGTT-3′ Mouse GAPDH: Forward 5′-AGTGTTTCCTCGTCCCGTAG-3′ and Reverse 5′-GCCGTTGAATTTGCCGTGAG-3′.

### 3.7. Statistical Analysis

The data are summarized as the means ± standard errors of the mean (SEMs). Statistical analyses performed are delineated in the figure legends. Post hoc comparisons of ANOVA results were corrected with the Tukey method. The Kolmogorov–Smirnov (KS) test or D’Agostino–Pearson normality test was used to determine whether the distribution of the data was consistent with a Gaussian distribution. Prism 5. software was used for statistical analyses. Statistical significance was accepted at *p*-values of <0.05.

## 4. Discussion

Trophoblast differentiation is a fundamental process ensuring successful placental development and function [2,3]. Altered placental differentiation and proliferation result in gestational vascular complications such as preeclampsia and intra-uterine growth restriction (IUGR) [8,10,14,19]. Procoagulant extracellular vesicles and platelets coordinately induce thrombo-inflammation, which may alter cellular function and ultimately result in organ dysfunction [20]. In pregnancy, they have been associated with placental pathologies, abortions, preeclampsia, and IUGR. Here, we show that EVs and platelets alter trophoblast cell differentiation and glycogen-rich cell content via the NLRP3 inflammasome.

The placental labyrinth, which starts developing after day 8.5 p.c., which constitutes the largest portion of the murine placenta, allows nutrient exchange to support fetal growth past day 10.5 p.c. [2,3,21]. The reduced labyrinthine development and Gcm-1 expression upon EV administration reflect a placental developmental delay in association with reduced syncytiotrophoblast differentiation. The junctional zone, which provides structural support between the invasive giant trophoblast and vascular syncytiotrophoblast, expresses angiogenic factors such as soluble VEGF receptor (sFlt-1) and proliferin-related protein (Plfr, originally called Prp) [22]. Correspondingly, we previously observed a correlation between the STC size and increased sFlt-1 in EV-injected mice [8], suggesting a causal link between altered trophoblast differentiation and increased sFlt-1. Future studies are needed to determine whether the increase in sFlt1 represents an increased number of STCs resulting in increased sFlt1 levels, or whether the cells themselves are dysfunctional and produce more sFlt1 per cell.

Interestingly, we observed increased GRCs in our model. The role of GRCs remains incompletely defined. An increase and a decrease in GRCs have been linked with impaired fetal development. The increase in glycogen-rich cells in our model may reflect an inability of these cells to metabolize glycogen to glucose, resulting in growth restriction. A specific population of GRCs has not been reported in humans but altered glycogen deposits in pregnancies complicated by preeclampsia have been reported [23]. Further studies exploring the mechanisms regulating glycogenesis, defective transport mechanisms, specific knockout mouse models, and single-cell sequencing approaches are required to better understand the role of placental glycogen for normal and impaired placental function in humans and in mice.

Besides the capability of EVs to activate thrombo-inflammation, they carry cargo such as mRNAs, miRNAs, and functionally active proteins. Therefore, they can convey different biological activities as mediators. Of note, small EVs can confer immune tolerance during pregnancy that aids the placenta in evading the maternal immune system. This suggests that EVs may have a physiologically beneficial role in pregnancy. However, we and others have shown a pro-inflammatory function of EVs during vascular complications such as PE. Placental-derived EVs are increased in maternal plasma in PE patients. Trophoblast-derived EVs during PE also possess reduced endothelial nitric oxide synthase (eNOS) and less nitric oxide (NO) activity and can potentially regulate endothelial function during pregnancy [24]. Therefore, besides the procoagulant activities, we do not exclude other functions of EVs. In our studies, we wanted to test the hypothesis that procoagulant EVs can modulate trophoblast differentiation by activating a thrombo-inflammatory pathway. Therefore, we focused on this particular aspect. Studies focusing on other aspects of EV source or function can help in identifying other roles of EVs in pregnancy. We speculate that EVs that reach the placenta release their cargo, which alters trophoblast differentiation and function. Likewise, poorly defined platelet-derived factors regulate placental function and trophoblast migration [25]. These factors may include TGF-β, ADP, thromboxane A2, and prostaglandins. Aberrant TGF-β signaling as well as thromboxane A2 is known to alter trophoblast differentiation and proliferation [26,27]. While prostaglandin E2 promotes trophoblast migration [28], its effects on trophoblast differentiation are unknown. A physiological function for platelet-derived factors, such as CCR1-ligands, has been proposed [29,30]. At the same time, both excess platelet activation and prostaglandins are known to promote inflammatory processes thereby preventing its resolution [29,31]. Likewise, inflammasomes have been suggested to convey physiological and pathophysiological effects during placentation, which may depend on the specific inflammasome activated, its magnitude, and timepoint [32]. These data support a concept in which platelets and inflammatory mechanisms, such as inflammasomes, convey homeostatic effects, which, however, need to be well-orchestrated during placental development. Therefore, undefined mechanisms, which may depend on other cell types (e.g., inflammatory cells), appear to contribute to the altered trophoblast differentiation and placental morphogenesis. In-depth insights into these mechanisms are required to allow developing therapies that target the harmful effects of thrombo-inflammation but preserve the physiological functions required for development.

## Supplementary Materials

The following are available online at https://www.mdpi.com/article/10.3390/ijms22189873/s1.
