# Supplementary material for: Procoagulant Extracellular Vesicles Alter Trophoblast Differentiation in Mice by a Thrombo-Inflammatory Mechanism
Source: Int J Mol Sci. 2021 Sep 13;22(18):9873. doi: 10.3390/ijms22189873 (PMC8466022; doi:10.3390/ijms22189873)
Supplement: Supplementary file 1 [file ijms-22-09873-s001.zip › ijms-1364605-supplementary.pdf]

Supplementary Table-S1: Patient Characteristics

	Controls (n=14)		PE (n=14)		
	**Mean**	**SD**	**Mean**	**SD**	***P* value**
**Age (years)**	34	4	31	5	0.08
**BMI (kg/m2)**	28	4	28	6	0.57
**Parity**					
**Primipara**	7 (50%)		10 (71.14%)		1
**Multipara**	7 (50%)		4 (28.57%)		
**Systolic blood pressure (mmHg)**	122.50	8.61	166.79	8.15	<0.001
**Diastolic blood pressure (mmHg)**	75.00	7.07	103.57	10.42	<0.001
**Proteinuria (g/24h)**	0.00	0.00	3995.07	4794.75	<0.001
**Gestational diabetes**	0	0	0	0	-
**Gestational age at birth**	40	1	36	4	0.01
**Relative birth weight (weeks)**	3447.14	295.48	2420.33	252.16	0.004
**Method of delivery**					
**Vaginal**	0	0	0	0	-
**C-section**	14	0	14	0	-
**Sex of child**					
**Boy**	7 (50%)		7 (50%)		1
**Girl**	7 (50%)		7 (50%)		
